# Improving regulatory capacity to manage risks associated with trade agreements

**DOI:** 10.1186/s12992-015-0099-7

**Published:** 2015-03-21

**Authors:** Helen L Walls, Richard D Smith, Peter Drahos

**Affiliations:** London School of Hygiene and Tropical Medicine, London, UK; Leverhulme Centre for Integrative Research on Agriculture and Health, London, UK; The Australian National University, Canberra, Australia; Queen Mary University of London, London, UK

**Keywords:** Trade policy, Trade agreements, Regulatory capacity, Health inequities

## Abstract

Modern trade negotiations have delivered a plethora of bilateral and regional preferential trade agreements (PTAs), which involve considerable risk to public health, thus placing demands on governments to strengthen administrative regulatory capacities in regard to the negotiation, implementation and on-going management of PTAs. In terms of risk management, the administrative regulatory capacity requisite for appropriate negotiation of PTAs is different to that for the implementation or on-going management of PTAs, but at all stages the capacity needed is expensive, skill-intensive and requires considerable infrastructure, which smaller and poorer states especially struggle to find. It is also a task generally underestimated. If states do not find ways to increase their capacities then PTAs are likely to become much greater drivers of health inequities. Developing countries especially struggle to find this capacity. In this article we set out the importance of administrative regulatory capacity and coordination to manage the risks to public health associated with PTAs, and suggest ways countries can improve their capacity.

## Main text

Trade agreements can lead to substantial benefits, including economic growth, lower cost and wider access to goods through trade barrier reductions, and reduced risk of military conflict between countries where their economies are more entwined [1]. However, trade liberalisation concerns the public health community, for example through the increasing spread of goods, people and services, and the contribution to social inequalities [2,3]. For example, risk commodity industries such as those for tobacco, alcohol and processed food are, via the commodities they produce, advertise and distribute, a key global driver of the growing burden of non-communicable disease (NCD), including diabetes, cardiovascular disease and cancer [2,4,5].

But the scope of international trade is fast changing. Whilst formerly preferential trade agreements (PTAs) were generally negotiated through the General Agreement on Tariffs and Trade, and later the World Trade Organization (WTO), there has been over the last two decades a proliferation of bilateral and regional PTAs with increasingly ‘deep’ commitments that go beyond those required by the WTO system. These commitments are sometimes called ‘WTO-plus’ (consistent with but that go beyond WTO agreements) and ‘WTO-X’ (outside the WTO framework) [6], and are often focused on reducing ‘behind the border’ operations, measures imposed internally in the domestic market to address, for example, health and environmental standards [7,8]. Thus modern PTAs, often better described as investment treaties, impact widely on areas of state regulation, including intellectual property (IP), investment, services, government procurement, technical standards and sanitary and phytosanitary standards. Of particular concern is the inclusion of the investor-state dispute settlement (ISDS) mechanism, allowing foreign companies to sue host governments for compensation when policy changes threaten their ability to generate earnings from investments, limiting signatory countries’ ‘policy space’ [9]. Such a mechanism is proposed in two key PTAs currently under negotiation, the Trans Pacific Partnership (TPP), a PTA involving twelve Asia-Pacific countries, Australia, Brunei, Canada, Chile, Japan, Malaysia, Mexico, New Zealand, Peru, Singapore, the United States and Vietnam [10], and the Transatlantic Trade and Investment Partnership (TTIP), between the European Union and the United States [9].

Expanded IP chapter provisions are also concerning, particularly with the potential for ‘evergreening’ of pharmaceutical patents, or the extension of patents on medicines for slight changes to formulation without the provision of superior benefit [11,12]. Other changes advocated in PTAs, for example proposals for an annex to the transparency chapter of the TPP endanger pharmaceutical coverage programmes through risks to effective pricing strategies such as therapeutic reference pricing, providing new avenues for industry to appeal decisions and requiring additional disclosure of information and avenues for consultation and input by the industry. Others have described more comprehensively these risks to pharmaceutical coverage and reimbursement programmes [13,14].

As articulated by WHO Director-General Margaret Chan [15] and others, such provisions in modern PTAs shift the balance of policy-making in favour of corporate interests, limiting policy options available to governments to protect public health [7,10], and lead to ‘policy chill’, with governments reluctant to legislate for public services for fear of lawsuits from foreign investors [9,16].

Examples of the public health implications of these new PTA provisions are already emerging. For example, the pharmaceutical company Eli Lilly is suing the Canadian government for CAD$500 million, under an ISDS mechanism in the North American Free Trade Agreement (NAFTA), for revocation of patents on two drugs which failed to show substantial benefit, even though the revocation of those patents was upheld in the courts [17,18]. Also using an ISDS mechanism, the tobacco company Philip Morris is, via a bilateral investment treaty with Hong Kong, demanding compensation from the Australian Government for the introduction of plain-packaged cigarettes [19]. There is emerging evidence of the impact the TPP may have on signatory countries, threatening affordability and access to medicines in New Zealand [13] and Vietnam [20], for example.

However whilst the concerns are well documented and examples of the implications emerging, there is a vacuum of literature on how decision-makers should respond to such threats. Creating stronger and more legally defensible general health exceptions within PTAs is an important public health target, and should be of highest priority. It is also a formidable challenge, not least due to inequalities in bargaining power between countries with the least and most to gain from these ‘intrusive’ PTA provisions, and the powerful industries backing some governments. Countries should give careful consideration as to whether it is in their interest to sign up to these PTAs at all. But such decision-making is often part of the negotiation process, in which to participate effectively requires a country have considerable administrative regulatory capacity, especially given the costs and benefits to becoming party to a PTA are often very difficult to assess.

Given the challenges of negotiating PTAs, and for those countries that do become signatories to PTAs, the on-going need for further PTA ‘risk management’, we have sought to identify the administrative regulatory capacities a country needs to develop to protect its national public health interests. We use the term administrative regulatory capacity to refer to a range of capacities. Administrative capacities include the capacity to implement procedures (for example, procedures of public consultation, coordination amongst government departments) and rules (for example, the rules required to register patents or implement food safety standards). Regulatory capacity relates to the technical competence required to interpret, monitor, adjust and, if necessary, set new regulatory standards. As we will see, a state needs deep reservoirs of these kinds of capacities if it is to protect its public health interests in the negotiation, implementation and management of a PTA. The capacity issues we identify pose the greatest problems for least-developed and developing countries, but are not confined to them. Even middle-sized developed countries experience dimensions of the capacity problems that we identify.

First, we address the importance of public sector administrative regulatory capacity and coordination for managing the risks to public health from PTAs within an evolving global trade system, and second, we suggest ways that countries can improve this capacity.

### Negotiation of PTAs

The negotiation of PTAs requires significant administrative regulatory capacity. But even then they should be approached with caution, given emerging evidence of the extreme risks of becoming party to the provisions in modern PTAs.

At the time of negotiation, it is often difficult to know the real costs and benefits of PTAs for public health because of the indeterminacy of the principles they establish or because of difficulties predicting the effects of specific rules in dynamically changing markets. An example of the indeterminacy problem is the current WTO litigation over Australia’s plain packaging legislation for tobacco products (a second legal challenge to Australia’s plain packaging legislation). During the 1970s and 1980s tobacco multinationals became advocates of stronger trademark protection. Both individually and as part of business organizations such as the International AntiCounterfeiting Organization, companies such as Philip Morris pushed an international trademark agenda. Briefly, stronger trademark protection became a key issue for a number of globalized industry sectors in the 1980s, leading a coalition of US, European Union and Japanese multinationals to draft text for an IP agreement [21]. In 1988 this coalition presented its draft to the main government players involved in negotiating what became the Agreement on Trade-Related Aspects of Intellectual Property Rights (TRIPS) (1994) [21]. Included in this draft was wording on the principle of the ‘unjustified encumbrance of a trade mark’, a variant of which is found in one of the articles central to TRIPS and now, some 25 years after its articulation by multinational business interests, the subject of WTO proceedings in which a number of countries are arguing Australia’s plain packaging law breaches the principle.

Understanding the full costs of TRIPS would have been difficult back in the 1980s. This is precisely the point. Trade negotiators did not understand them and most likely made little attempt to do so. This example serves as a warning to today’s trade negotiators, and highlights the danger of entering into an agreement where business interests have a disproportionate influence on the PTA text. Where business interests do have a disproportionate influence on the PTA text, states could easily leave the negotiating table having delivered private gains to industry actors while increasing costs to the public in the form of, for example, higher prices to medicines.

The risk management problem in regard to the negotiation of PTAs is exacerbated for developing countries by an inequality of bargaining power. PTAs involving the US, for example, are by far the most comprehensive in terms of their coverage of goods and services and their inclusion of WTO-plus provisions with greater public health risk [22]. Studies of PTAs between large developed countries and small developing countries find a weak reciprocity; the small country gives up a lot to gain a little [23]. Importantly, the developing country in these cases takes on developed country regulatory standards without having the capacity of the developed country to manage the risks of those standards [24].

An example of this relates to food safety standards. Through agreements such as WTO’s Sanitary and Phytosanitary (SPS) Agreement, developing countries have been drawn into complying with international standards set by bodies such as the Codex Alimentarius Commission. Some PTAs under negotiation include provisions relating to SPS standards, that are ‘WTO-plus’ and ‘WTO-X’ [6]). Most developing countries lack the scientific capacity needed to evaluate the public health costs and benefits of these food standards or to play an influential role in the technical meetings that produce them [25-27].

### Implementation and on-going management of PTAs

Not only do countries need the administrative regulatory capacity to effectively negotiate PTAs, if they do decide to become signatories, they also need to increase their capacities in regard to PTA implementation and on-going management. The earlier examples of corporations suing the Australian and Canadian governments highlight the need for administrative capacity to defend public interests against globally coordinated litigation strategies backed by multinationals, law firms and lobbyists [11,28,29].

On-going management of PTAs poses a huge administrative burden to governments. For example, PTAs often create a linkage between a drug registration authority’s work and the patent system by requiring the drug authority to establish procedures that allow a patent owner to prevent the marketing of a drug by a third party that it believes affects its patent rights [30]. This requires independent scrutiny of a patent owner’s strategic use of patents. The United States has the Federal Trade Commission and the Antitrust Division of the Department of Justice to police anticompetitive uses of IP by pharmaceutical companies, with budgets and full-time equivalent staff (2013) of US $312 million and 1176, and US$165 million and 851, respectively [31-33] – a scale out of most countries’ reach.

Kesselheim et al. [29] have described the pharmaceutical industry’s systematic engagement in activities (legal and illegal) to promote drug sales and their resistance to external regulatory approaches. The US Attorney General has noted the difficulty of enforcing limits on pharmaceutical industry behaviour in managing the risks of monopoly extension, despite the considerable resources available to the US government [11].

### States need to find ways to increase administrative regulatory capacity

If states do not find ways to increase their administrative regulatory capacities in regard to the negotiation, implementation and on-going management of PTAs, these PTAs will potentially drive greater health inequities [10,34]. But how might countries, and particularly small and poor countries, improve their capacity to manage the risks of PTAs? The scale of the problem is daunting, however we offer the following suggestions.

First, states have to recognize that PTAs carry social risks and costs, and that administrative regulatory capacity is needed for their risk management, including their negotiation, and later, assuming a country does choose to become a signatory, implementation and on-going management.

Second, states should not confuse this risk management task with compliance and acquiescing to donor aid objectives. The EU, for example, has been active in Southeast Asia helping countries establish patent offices. However, the grant of patents by these offices overwhelmingly benefits EU companies in sectors such as pharmaceuticals. Accepting donor packages for patent offices that do not deal with well-known problems such as ‘evergreening’ of pharmaceutical patents, will simply increase rather than decrease risks to public health [35]. Whilst external assistance can be useful and non-governmental organisations supporting developing countries often play a crucial role here, the politics of ‘aid for trade’ is complex [36,37], and there is danger in accepting institutional aid from regulatory missionaries with compliance objectives.

Third, since resources are scarce, developing countries should pick administrative regulatory capacity targets, giving priority (considering limited resources) to creating nodes of excellence – areas in which the development of regulatory capacity is prioritised – in public health regulation, rather than attempting with limited resources to achieve such regulatory ‘excellence’ across the board of needed regulation, which given resource constraints may be unrealistic. It may be prudent to develop a node of excellence first in regard to the negotiation of PTAs, however, and this relates to point five, if such expertise can be appropriately shared perhaps regionally, a different regulatory target may be more appropriate.

A fourth and related point is that creating these nodes of excellence might be easier and indeed more justifiable by learning from the regulatory experience of other countries and encouraging the dissemination of best practice. Such regulatory learning from others’ examples is difficult, being more bounded than rational and subject to biases [38]. Others’ adoption of a seemingly successful practice can be highly motivating. But it is also the case that developing countries such as Brazil, China and India have begun to realize the problems of trade agreements containing IP chapters and have started to address these through regulation. For example, Brazil has devised an approach in which the National Sanitary Surveillance Agency (ANVISA) is involved in the examination and grant of pharmaceutical patents rather than having all regulatory power over the grant of pharmaceutical patents centred in the Brazilian patent office [39]. This model of more disperse regulatory power grants some influence to a public health perspective rather than simply paying it lip service. It also provides a safeguard against regulatory capture (when a regulatory agency tasked with acting in the public interest instead advances the commercial or special concerns of interest groups that dominate the industry of sector it is charged with regulating) of a patent office. In a world where governance and regulation increasingly takes on network forms, Brazil’s experiment with ANVISA forms a node of excellence with which other developing countries might form ties. These might range from sharing experiences and data to adopting some variant of the ANVISA model. In regard to food trade and nutrition, inspiration and learning may be drawn from, for example, Ghana’s innovative use of food standards to reduce the availability of high-fat meat (mainly imported) from the national food supply, but in compliance with international trade law [40].

Fifth, developing countries specifically should be looking to intensify collaboration and networking amongst themselves. Some authors have documented an increasing interest in such ‘south-south cooperation’ [41]. Hoekman et al. [42] has suggested a ‘services knowledge platform’ to bring together regional regulators, officials and stakeholders to discuss regulatory reform for trade and investment services. In Southeast Asia, the Association of Southeast Nations (ASEAN) has been considered a platform for such regional cooperation for trade and health [2]. Rather than trying to achieve a ‘regulatory state’ involving governmental structures, developing countries might instead work towards a ‘regulatory society’ of networked governance in which to achieve regulation, with the state networked to non-state actors such as non-governmental organisations, industry co-regulators, professional and international organisations [43]. US models of private enforcement of public regulation depend on an entrepreneurial adversarial legal culture that is not found in most developing countries. An African country is just as likely to gain valuable insights from studying the South African Competition Commission’s experience in regulating pharmaceutical companies as it is from studying the work of the US Federal Trade Commission [20]. South-South regulatory learning and diffusion – and indeed, coordinated action – is just as important as North-South learning in this context.

## Conclusion

Entering into PTAs should be approached cautiously, and with the capacity to negotiate effectively. However for those states that do decide to proceed, it is important to have the capacity to manage the risks they pose to public health through regulation. Thus, the negotiation, implementation and on-going management of PTAs all require considerable administrative regulatory capacity – a significant administrative burden on governments. States must recognize the importance of this risk-management task; distinguish it from regulatory compliance; and focus on regulatory learning and building nodes of excellence in regulation. Developing countries especially will benefit from networking to overcome capacity deficits and look to South-South collaborations in the process of regulatory learning.

## Abbreviations

ANVISA: National Sanitary Surveillance Agency of Brazil
IP: Intellectual property
PTAs: Preferential trade agreements
TRIPs: Agreement on Trade-Related Aspects of Intellectual Property Rights
WTO: World Trade Organization
WTO-plus: Provisions in preferential trade agreements that go further than those in existing WTO agreements
WTO-X: Provisions in preferential trade agreements that are outside the scope of existing WTO agreements
